# Obesity in Pregnancy as a Risk Factor in Maternal and Child Health—A Retrospective Cohort Study

**DOI:** 10.3390/metabo14010056

**Published:** 2024-01-15

**Authors:** Miriam Orós, Marta Lorenzo, María Catalina Serna, Júlia Siscart, Daniel Perejón, Blanca Salinas-Roca

**Affiliations:** 1 Family Medicine Department, University of Lleida, 25003 Lleida, Spain; jvsiscart.lleida.ics@gencat.cat (J.S.); dperejon.lleida.ics@gencat.cat (D.P.); 2 Miami Platja Health Center, Catalan Institute of Health, 43892 Tarragona, Spain; 3 Eixample Health Center, Institut Català de la Salut, 25003 Lleida, Spain; 4 School of Medicine, Lleida University, 25003 Lleida, Spain; 5 Serós Health Center, Catalan Institute of Health, 25183 Lleida, Spain; 6 Cervera Health Center, Catalan Institute of Health, 25200 Lleida, Spain; 7 Grow-Global Research on Wellbeing (GRoW) Research Group, Blanquerna School of Health Science, Ramon Llull University, Padilla, 326–332, 08025 Barcelona, Spain

**Keywords:** obesity, pregnancy, overweight, complications

## Abstract

The prevalence of overweight and obesity has risen dramatically in the last few years. This has led to an increase in both conditions in pregnant women. Obesity and overweight are associated with complications for both the mother and the newborn. The aim of this study is to determine the prevalence of obesity and its association with the risk of complications during pregnancy. Materials and Methods: We conducted a retrospective cohort study of pregnant women who delivered from 1 January 2012 to 31 December 2018. Results: A higher prevalence of obesity is observed in the group of women aged 35 or older. Women with a BMI > 25 present a higher risk of cesarean section (aOR 1.49, 95% CI: 1.37–1.61), preeclampsia (aOR 1.64, 95% CI: 1.19–2.26), high-risk pregnancy (aOR 2.34, 95% CI: 1.68–2.6), Apgar < 7 at one minute (aOR 1.53, 95% CI: 1.25–1.89) and macrosomia (aOR 2.08, 95% CI: 1.83–2.37). Maternal overweight and obesity are important determinants of the risk of complications for both the mother and the newborn.

## 1. Introduction

In recent decades, the prevalence of overweight and obesity (established as BMI 25–30 and BMI ≥ 30 kg/m^2^, respectively) has risen to epidemic proportions. In pregnant women, the prevalence of both conditions has also increased dramatically in both high- and middle-income countries [1], but the exact burden of overweight and obesity during pregnancy remains unclear. Some studies suggest that in 2025, more than 25% of all women in the world will suffer from obesity [2].

In Europe, estimations of the prevalence of overweight and obesity are alarming, given that most countries report figures approaching 20% in the general population [3]. In pre-pregnant women in Europe, the rates range between 26.8% and 54% [4]. In Spain, around 30% of women are overweight, and as many as 16.7% are obese [5].

Obesity during pregnancy is associated with short and long-term complications for both the mother and the newborn, such as gestational hypertension and diabetes, preeclampsia, preterm birth and increased risk of miscarriage or congenital defects [1]. Furthermore, obese pregnant women with a previous diagnosis of other diseases (such as diabetes or hypertension) have a higher risk of complications during pregnancy and childbirth [2]. For their part, children born to mothers with obesity have a greater risk of being overweight or obese both early in life and at later stages [6]. The epigenetic programming that occurs at conception and during pregnancy may predispose these children to various chronic metabolic conditions [7].

The aim of the present study was to evaluate the consequences of maternal obesity on pregnancy, childbirth and the state of the newborn in the health region of Lleida, Spain, over a 7-year period.

## 2. Materials and Methods

### 2.1. Study Design and Data Collection

From 2012 to 2018, pregnant women from the health region of Lleida participated in a retrospective observational cohort study.

Information on women who had delivered at the Arnau de Vilanova Hospital between 1 January 2012 and 31 December 2018 was obtained from the CMBD database, which compiles data on hospital discharges. Clinical histories were retrieved from the E-CAP database of the Catalan Institute of Health (ICS) for all eligible patients allocated to the primary care area, and data related to health prescriptions were also collected from the ICS database.

This article is part of the Iler Pregnancy Project carried out in the health region of Lleida, which was designed in order to evaluate the prevalence of chronic pathologies such as depression, obesity, diabetes mellitus and hypothyroidism during pregnancy and to assess adherence to the medication prescribed [8].

### 2.2. Study Population

All women who gave birth between 1 January 2012 and 31 December 2018 at the Arnau de Vilanova Hospital (HUAV) were included in this study. Patients who were not assigned to the area of Lleida or who were attended privately were excluded. Data were included from the date of last period until delivery (therefore, some data collected are from 2011 in the cases of women whose last period occurred before 2012).

To assess the representativeness of the sample, the number of births in our study was calculated as a percentage of the total number of births in the health region, extracted from the database of the Statistical Institute of Catalonia (Idescat) (Table 1).

Indicators under review were the following:

- Perinatal: Age; body mass index (BMI) in kg/m^2^: normal weight (BMI between 18.5 and 24.9 kg/m^2^), overweight (BMI between 25 and 29.9 kg/m^2^) and obesity (BMI ≥ 30 kg/m^2^) [9]; number of pregnancies and multiple pregnancies; diabetes mellitus (code O24.9 in ICD-10); hypothyroidism (code EO3.9 and EO2 in ICD-10); arterial hypertension (code I10-I16 in ICD-10); dyslipidemia (code E78 in ICD-10); depression (codes F32.0-F32.9, F33.0-F33.3, F33.8, F33.9, F34.1, or F41.2 in ICD-10); and preeclampsia and level of risk during pregnancy.
- Childbirth: Method of delivery (vaginal delivery vs. caesarean section); preterm birth (<37 weeks of pregnancy) or prolonged.
- Neonatal: Birth weight (underweight < 2500 g, normal weight 2500–4000 g and macrosomia > 4000 g); Apgar test score at 1 min and at 5 min (good Apgar score if ≥7 points, poor Apgar score < 7 points).

### 2.3. Data Analysis

A descriptive analysis was performed using absolute and relative frequencies for categorical variables and means and standard deviations for numerical variables. To assess the differences between groups, the Chi-squared test was used for categorical variables and the Student *t*-test for numerical variables.

Multivariate logistic regression was used to assess the association between obesity and overweight and the perinatal, labour and neonatal complications, calculating odds ratios (OR) with the respective 95% confidence intervals. Statistical significance was defined as *p* < 0.05.

### 2.4. Ethical Aspects

This study received approval from the ethics and clinical research committee of the Jordi Gol Primary Care Research Institute (IDIAP), on 29 April 2020, code no. 19/195-P, and was executed in accordance with the tenets established in the Declaration of Helsinki. Data were obtained from electronic medical reports recorded in the E-CAP database and were collected by the Department of Health Evaluation and Research Management Unit. Informed consent was not required. Furthermore, the information from E-CAP was processed anonymously and confidentially as required by national legislation and in Regulation 2016/679 of the European Parliament and of the Council for the protection of individuals.

## 3. Results

**Figure 1:**
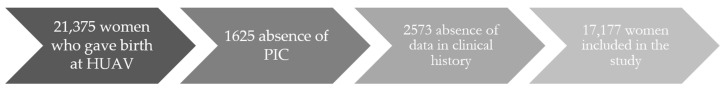
Study sample.

The initial sample comprised 21,375 women who had delivered a child at the HUAV from 2012 to 2018. Of these, 1625 patients were not included due to the absence of a personal identification code (PIC), and 2573 others were excluded because of the absence of data in their clinical history. The final sample thus comprised 17,177 patients (Figure 1).

Table 2 shows that the presence of obesity and overweight was significantly affected by maternal age. Obesity was present in 10.7% of pregnant women under the age of 30 and in 13.2% of those aged 35 or older. Regarding the relationship between BMI and maternal complications, preeclampsia was recorded in 1.7% of patients compared to a rate of 0.8% in patients with BMI < 25 kg/m^2^. As for the effects on the neonate, caesarean delivery was performed in 15.3% of women with normal weight compared to a rate of 26.4% in the group with BMI > 30 kg/m^2^. Additionally, the percentage of pregnancies classified as high risk was 1.8% in patients with BMI < 25 as opposed to 3.3% in the group with BMI > 30.

In reference to perinatal outcomes, fetal macrosomia was recorded in 5.1% of neonates born to women with normal weight in contrast to 11.4% of those born to women with overweight. Apgar scores one minute after birth were below 7 in 2.2% of neonates born to mothers with BMI < 25 compared to 4% in those born to mothers with BMI > 30 prior to pregnancy, and Apgar scores at five minutes were below 7 points in 0.6% of neonates of women with BMI < 25 and in 1.3% of those born to women with BMI > 30.

**Figure 2:**
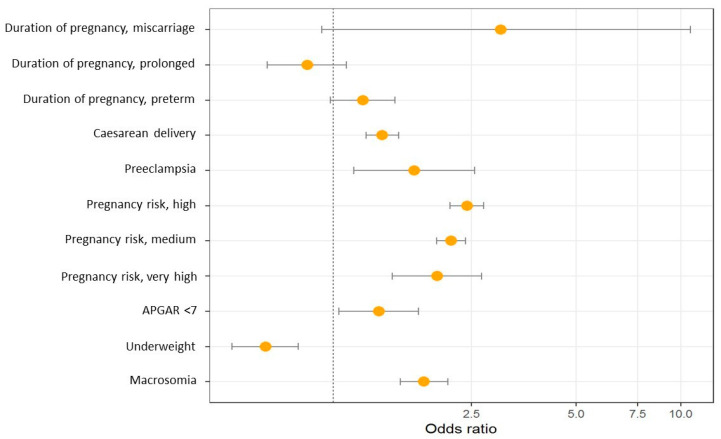
Odds ratio graph between obstetric characteristics and maternal BMI.

Figure 2 and Table 3 show the results of the multinomial logistic regression between obstetric characteristics and maternal BMI. Pre-pregnancy obesity is associated with an increased risk of caesarean delivery, preeclampsia, higher risk during pregnancy, macrosomia and lower Apgar score.

## 4. Discussion

The results of this retrospective cohort study show that the prevalence of pre-pregnancy overweight and obesity in pregnant women is 22% and 11.7%, respectively. Women with pre-pregnancy obesity presented a higher risk of maternal and neonatal complications, including caesarean delivery (aOR 1.49, 95% CI: 1.37–1.61), preeclampsia (aOR 1.64, 95% CI: 1.19–2.26), higher risk pregnancy (aOR 2.34, 95% CI: 1.68–2.6), Apgar score < 7 at one minute (aOR 1.53, 95% CI: 1.25–1.89) and macrosomia (aOR 2.08, 95% CI: 1.83–2.37). In contrast, women with obesity had a lower risk of low birth weight (aOR 0.63, 95% CI: 0.51–0.79).

González-Plaza et al. [10] reported pre-pregnancy overweight in 18.9% of pregnant women in their study and obesity in 8.4%, while Melchor et al. [5] recorded respective figures of 25% and 13.3%, similar to those presented in our study. The European Perinatal Report found that Poland (7.1%), Slovenia (9.0%) and France (9.9%) had the lowest levels of obesity in pregnant women, while Scotland (20.7%) had the highest. Most other European countries had figures between 12 and 14%, which is in line with our study [11].

The risk of complications during pregnancy is high. Kim et al. [12] estimated the overall risk of complications in obese women to be between 18% and 47%. The meta-analysis carried out by Santos et al., with individual data from 39 cohorts, estimated that maternal overweight/obesity accounted for 23.9% of any pregnancy complication [13]. A retrospective cohort study using publicly available data from a high-income population found that the risk of fetal, neonatal or infant death increased with maternal BMI both above and below the 21–22 kg/m^2^ range, which is the lowest risk [14].

González-Plaza et al. [10] reported a higher frequency of maternal complications such as preeclampsia and gestational diabetes in overweight and obese pregnant women. Similar results have been published by other studies [8,15,16,17]. In an analysis of complications related to the degree of obesity, Kim et al. [12] found a four-fold increase in preeclampsia in women with grade III obesity compared to those with normal weight. Finally, a historical cohort study analysing the Spanish population reported a higher risk of preeclampsia in obese than in normal-weight women (aOR 2.199, 95% CO: 1.46–3.29) [18]. These data are consistent with the results observed in our study.

In the study by Sunder et al. [19], obese women were older, more likely to be multiparous and had a higher rate of spontaneous abortions than non-obese women. Our results do not show a significant increase in miscarriage among women with obesity, but there was a greater association with age and multiparity.

In our study, women with BMI > 25 were significantly more likely to be classified as high-risk pregnancies compared with those of normal weight. Our results corroborate those recorded in the study by Melchor et al. [5] (aOR 2.755, 95% CI: 2.46–3.08). Furthermore, weight gain during pregnancy by overweight or obese women could result in aggravating conditions for developing non-communicable diseases.

González-Plaza et al. [10] reported cesarean section in 43.9% of obese women, a percentage higher than our rate of 26.4%. Other studies record figures that range between 18.7% and 35% [20]. An analysis of the possible adverse effects of obesity and gestational diabetes mellitus showed that both (either alone or combined) were associated with a higher rate of macrosomia and a higher rate of caesarean delivery [21]. That study recorded a progressive increase in neonatal weight in the following order: lowest weight in the group without obesity or gestational diabetes; second lowest in the group with gestational diabetes but no obesity; second highest in patients with obesity but no gestational diabetes; and highest in the group with both obesity and gestational diabetes. These results may account for the relatively low percentage of underweight newborns in our study compared with previous reports [22].

Obesity is known as determinant for failured induction of labor, for emergency caesarean section and for perinatal complications [15,23,24,25]. Zhu et al. [26] reported a higher risk of Apgar score below 7 at one minute after birth in newborns of overweight women. This systematic review also demonstrated associations between Apgar < 7 at 5 min and maternal BMI: overweight (OR 1.13; 95% CI, 1.08–1.20), obesity (OR 1.40; 95% CI, 1.27–1.54) and morbid obesity (OR 1.71; 95% CI, 1.55–1.89). No association was observed between maternal BMI and umbilical cord pH. Our study showed an increased risk of Apgar < 7 at both one and five minutes.

A national analysis of preterm infants by a Swedish study demonstrates a correlation between the incidence of severe neonatal hypoxia and the mother’s rising BMI. Mothers with BMI > 35 presented a higher risk of low Apgar scores at 5 and 10 min, neonatal seizures and intraventricular haemorrhage grade 1–4 compared to those with BMI < 25. A greater risk of low Apgar scores at 5 and 10 min was also associated with the group of BMI 30 to 35 [27]. In a retrospective cohort study conducted in the USA with a population of 9,282,486 pairs of mothers and children, an increased risk of low Apgar score was also observed in cases of overweight (OR (95% CI) of 1.12 (1.11–1.14)), as well as in grade 1 obesity (1.21 (1.19–1.23)), grade 2 obesity (1.34 (1.31–1.36)) and grade 3 obesity (1.55 (1.51–1.58)) [28].

In another study by Vinturache et al. [29], an increased risk of macrosomia was observed in children of overweight and obese women compared to those born to women of normal weight. Similar results were reported by Gaudet et al. [30], with a prevalence of fetal macrosomia of 15.8% compared to 9.3% in those born to mothers with normal weight [31].

The results of a meta-analysis including 21 studies found that pre-pregnancy overweight and obesity increased the risk of macrosomia (OR, 1.67; 95% CI, 1.42–1.97; and OR, 3.23; 95% CI, 2.39–4.37) and subsequent offspring overweight and obesity (OR, 1.95; 95% CI, 1.77–2.13; and OR, 3.06; 95% CI, 2.68–3.49) [32]. Maternal overweight/obesity and gestational diabetes mellitus were considered in different studies as the main determinants of fetal macrosomia [33]. In a study that evaluates the possible influence of gestational diabetes mellitus as a potential mediator, the risk of macrosomia in overweight women is estimated at 40% [34].

We found that maternal pre-pregnancy overweight and obesity only increased the odds of macrosomia rather than low birthweight (aOR 0.63, 95% CI: 0.51–0.79), which was consistent with the conclusions of some meta-analyses [32,35]. Unlike our results, in other studies, both macrosomia and low birth weight of the newborn are related to maternal obesity [35,36], and large-for-gestational-age or small-for-gestational-age is also related to maternal obesity [37]. Different characteristics of the population, sample size or different covariates may account for the differences observed in our study. A prospective Polish cohort of 912 pregnant women was conducted, and newborn weight was analysed based on pre-pregnancy body mass index (BMI). Compared to women with normal BMI, women with maternal obesity were at a three-fold higher risk of macrosomia (AOR = 3.21 (1.69–6.1), *p* < 0.001). In this study, the prevalence of macrosomia was 10.6%, higher than the prevalence of low birth weight, which was found to be 6.6%. Higher maternal BMI values were associated with macrosomia, low birth weight and fetal growth restriction [22].

The mechanisms that cause complications in obese mothers during pregnancy are not well known. Factors such as endothelial dysfunction, lipotoxicity, inflammation or infection, insulin resistance or oxidative stress have been explored [23,38]. The excessive fat accumulation in obesity is caused by an energy imbalance that generates signalling through the TNF family cytokines that mediate cell death and inflammation within adipose tissue, eventually resulting in lipid leakage, glucotoxicity and insulin resistance [39]. In addition, elevated levels of these mediators (such as tumour necrosis factor or interleukin 6) cause placental inflammation, with detrimental effects on fetal metabolism, and increase the risk of metabolic syndrome and obesity in the long term [40].

The limitations of this study include the absence of data regarding obesity risk factors such as diet, physical activity or the social environment, which would have allowed us to refine our analysis. Other maternal factors that were not included in the study but might have influenced the gestational outcomes would be age, race or ethnicity, gestational weight gain or multiparity.

Another limitation of this study is the lack of information on some of the participants in our study, for example, newborns who were not weighed or Apgar-tested or women whose pregnancies were privately monitored. However, due to the Spanish National Health System’s universal coverage, the estimated representation of these participants is approximately 2.2% of the total. Therefore, they are unlikely to have influenced the study results.

## 5. Conclusions

Maternal obesity prior to pregnancy is associated with an increase in complications for both the mother and the newborn. Preventive strategies for promoting a healthy BMI before pregnancy can help to reduce the burden of complications in pregnancy and, ultimately, the morbidity for the mother and the newborn. Detecting overweight and obesity prior to pregnancy can help to implement health strategies, such as advising on healthy lifestyles or appropriate levels of physical activity, with interventions adapted to each individual characteristic.

Further studies are needed to evaluate public health and healthcare interventions that can help women achieve optimal health conditions during pregnancy and postpartum and thus prevent complications. Moreover, additional studies should be carried out to investigate the multifactorial origin of obesity and all the variables that may influence body weight, such as socio-demographic factors, religion or lifestyle.

## Figures and Tables

**Table 1:** Number of births registered in our sample compared to the total in the area of Lleida over the study period (by year).

Year	Births in Our Sample	Births Recorded in Idescat	Sample/Idescat
2012	3635	3788	96%
2013	3370	3535	95%
2014	3308	3592	92%
2015	3162	3426	92%
2016	3180	3283	97%
2017	3034	3197	95%
2018	3001	3029	99%

**Table 2:** Obstetric and socio-demographic characteristics and their association with pre-pregnancy BMI.

	BMI ≤ 25	BMI > 30	BMI 26–30	*p* Value
	N = 11,117	N = 1986	N = 3700	
Maternal age (years)				<0.001
Age < 30	4722 (69.1%)	734 (10.7%)	1381 (20.2%)	
Age 30–35	2925 (67.0%)	513 (11.8%)	927 (21.2%)	
Age ≥ 35	3470 (62.0%)	739 (13.2%)	1392 (24.9%)	
Number of pregnancies				<0.001
>4	194 (42.8%)	111 (24.5%)	148 (32.7%)	
1	6348 (71.9%)	838 (9.49%)	1645 (18.6%)	
2	3237 (63.9%)	616 (12.2%)	1213 (23.9%)	
3	1013 (55.4%)	311 (17.0%)	505 (27.6%)	
4	325 (52.1%)	110 (17.6%)	189 (30.3%)	
Preeclampsia				<0.001
No	11,037 (66.3%)	1952 (11.7%)	3660 (22.0%)	
Yes	80 (51.9%)	34 (22.1%)	40 (26.0%)	
Multiple pregnancies				1.000
No	11,094 (66.1%)	1982 (11.8%)	3696 (22.0%)	
Yes	23 (74.2%)	4 (12.9%)	4 (12.9%)	
Caesarean delivery				<0.001
No	9412 (67.8%)	1461 (10.5%)	3001 (21.6%)	
Yes	1705 (58.2%)	525 (17.9%)	699 (23.9%)	
Duration of pregnancy				0.528
Miscarriage	357 (64.4%)	74 (13.4%)	123 (22.2%)	
Prolonged	218 (67.1%)	38 (11.7%)	69 (21.2%)	
Preterm	479 (63.4%)	101 (13.4%)	176 (23.3%)	
On term	7427 (66.5%)	1296 (11.6%)	2448 (21.9%)	
Level of risk				<0.001
High risk	1566 (54.8%)	573 (20.1%)	718 (25.1%)	
Medium risk	2598 (58.2%)	847 (19.0%)	1018 (22.8%)	
Very high risk	177 (57.1%)	60 (19.4%)	73 (23.5%)	
No risk	5694 (75.7%)	330 (4.39%)	1501 (19.9%)	
Hypothyroidism				0.318
No	10,402 (66.3%)	1841 (11.7%)	3449 (22.0%)	
Yes	715 (64.4%)	145 (13.1%)	251 (22.6%)	
Hypertension				<0.001
No	10,986 (66.9%)	1841 (11.2%)	3585 (21.8%)	
Yes	131 (33.5%)	145 (37.1%)	115 (29.4%)	
Gestational Diabetes Mellitus				<0.001
No	10,616 (67.7%)	1715 (10.9%)	3358 (21.4%)	
Yes	501 (45.0%)	271 (24.3%)	342 (30.7%)	
Dyslipidemia				0.027
No	11,013 (66.3%)	1956 (11.8%)	3652 (22.0%)	
Yes	104 (57.1%)	30 (16.5%)	48 (26.4%)	
Depression				0.150
No	10,851 (66.3%)	1924 (11.7%)	3603 (22.0%)	
Yes	7 (0.83%)	19 (2.65%)	1 (0.45%)	
Birth weight (categorical variable)				<0.001
Underweight	604 (67.9%)	90 (10.1%)	195 (21.9%)	
Macrosomia	505 (50.1%)	198 (19.7%)	304 (30.2%)	
Normal weight	8749 (67.5%)	1447 (11.2%)	2762 (21.3%)	
Apgar 1 min (categorical variable)				<0.001
Apgar score ≥ 7	9621 (66.6%)	1659 (11.5%)	3162 (21.9%)	
Apgar score < 7	212 (57.8%)	70 (19.1%)	85 (23.2%)	
Apgar at 5 min (categorical variable)				0.014
Apgar score ≥ 7	9772 (66.5%)	1706 (11.6%)	3219 (21.9%)	
Apgar score < 7	63 (55.3%)	22 (19.3%)	29 (25.4%)	

**Table 3:** Multinomial logistic regression between obstetric characteristics and maternal BMI.

	OR	CI 2.5	CI 97.5	*p*-Value
Duration of pregnancy: miscarriage	3.0316031	0.9291086	10.6471137	0.068
Duration of pregnancy: prolonged	0.8438022	0.6460084	1.0938789	0.206
Duration of pregnancy: preterm	1.2177550	0.9827944	1.5068656	0.071
Caesarean delivery	1.3862618	1.2439709	1.5442462	<0.001
Preeclampsia	1.7122792	1.1466166	2.5493857	0.008
Pregnancy risk: high	2.4275486	2.1710439	2.7143552	<0.001
Pregnancy risk: medium	2.1809652	1.9830158	2.3989355	<0.001
Pregnancy risk: very high	1.9914195	1.4776223	2.6713605	<0.001
APGAR < 7 at 1 min	1.3535583	1.0380135	1.7602681	0.025
Underweight	0.6399526	0.5131872	0.7947356	<0.001
Macrosomia	1.8261564	1.5604792	2.1364279	<0.01

## Data Availability

The data used in this study are only available for the participating researchers, in accordance with current European and national laws. Thus, the distribution of the data is not allowed. However, researchers from public institutions can request data from SIDIAP. Further information is available online (https://www.sidiap.org/index.php/en/solicituds-en (accessed on 11 January 2024)).
